# Chronic Disease Onset Among People Living with HIV and AIDS in a Large Private Insurance Claims Dataset

**DOI:** 10.1038/s41598-019-54969-3

**Published:** 2019-12-06

**Authors:** Hsin-Yun Yang, Matthew R. Beymer, Sze-chuan Suen

**Affiliations:** 1 Department of Pharmaceutical and Health Economics, School of Pharmacy, University of Southern California, Los Angeles, USA; 2 Department of Community Health Sciences, Jonathan and Karin Fielding School of Public Health, University of California, Los Angeles, Los Angeles, USA; 3 Daniel J. Epstein Department of Industrial and Systems Engineering, Viterbi School of Engineering, University of Southern California, Los Angeles, USA

**Keywords:** Risk factors, HIV infections, Epidemiology

## Abstract

People living with HIV/AIDS (PLWHA) have a growing life expectancy in the US due to early provision of effective antiretroviral treatment. This has resulted in increasing exposure to age-related chronic illness that may be exacerbated by HIV/AIDS or antiretroviral treatment. Prior work has suggested that PLWHA may be subject to accelerated aging, with earlier onset and higher risk of acquiring many chronic illnesses. However, the magnitude of these effects, controlling for chronic co-morbidities, has not been fully quantified. We evaluate the magnitude of association of HIV infection on developing chronic conditions while controlling for demographics, behavioral risk factors, and chronic comorbidities. We compare chronic disease risks of diabetes, hypertension, stroke, cancers, lung diseases, cardiovascular diseases, and cognitive impairment between PLWHA and HIV- individuals in a large, de-identified private insurance claims dataset (~24,000 PLWHA) using logistic regressions. HIV status is statistically significantly associated with higher levels for all chronic illnesses examined, a result which is robust to multiple model specifications and duration of analysis (2, 5, and 10 years from enrollment). Our results suggest that PLWHA may be at elevated risk for a wide variety of chronic illnesses and may require additional care as the aging PLWHA population grows.

## Introduction

The life expectancy of people living with HIV/AIDS (PLWHA) has significantly increased due to the development of antiretroviral therapy (ART) in the mid-1990s. PLWHA who are treated with ART can now expect to live into their seventies, provided they are adherent to their ART regimen^1–3^. According to the US Centers for Disease Control and Prevention, almost half (47%) of PLWHA in the US were aged 50 and older in 2015; 18% were aged 60–64 and 16% were aged 65 and older^4^. The age distribution of PLWHA suggests that the US now faces the challenge of a growing population of aging PLWHA^5^. As the aging population of PLWHA increases, chronic diseases that are common among elderly populations play a more important role than ever before in the health care management of PLWHA. Moreover, these chronic diseases, including diabetes, hypertension, stroke, lung diseases, cancers, cardiovascular diseases, and neurocognitive disorders, occur more often in PLWHA than in people without HIV/AIDS^6–10^.

Although the mechanism by which HIV/AIDS is related with chronic diseases is not fully understood, researchers have found some possible explanations for the high prevalence of the chronic diseases seen in PLWHA. Among the seven aforementioned chronic diseases, diabetes, hypertension, stroke, and cardiovascular diseases are closely associated with metabolic syndrome – which includes abdominal obesity, atherogenic dyslipidemia, raised blood sugar, insulin resistance, proinflammatory and prothrombotic states^11–13^–which is one of the adverse side effects of antiretroviral therapy^14–19^. Other chronic diseases may be linked to long-term inflammation due to persistent low-level viremia that may occur even with ART^20^. Inflammation caused by HIV, independent of ART status, is associated with obstructive lung disease and cancer^20^. It is also associated with HIV-associated neurocognitive disorder (HAND)^21^, which is a collective term for HIV-associated dementia, HIV-associated mild neurocognitive disorder, and asymptomatic neuropsychological impairment. The high prevalence of these chronic diseases in PLWHA not only complicates the health management of PLWHA but may also pose a burden to the healthcare system^22–27^.

Existing studies have previously examined chronic illness prevalence among PLWHA in Medicare and younger cohorts in the United States^9,20,28,29^, as well as in populations in other countries. Here, we examine a population starting at age 50 using a private insurance claims dataset.

It is important to control for chronic comorbidities – a feature that prior studies have typically not examined – as many chronic conditions may interact. For instance, diabetes may elevate the risk of cardiovascular disease; chronic illnesses can exacerbate cognitive impairment or vice versa^30,31^. We therefore examine diabetes, hypertension, stroke, cancers, lung diseases, cardiovascular diseases, and cognitive impairment/dementia in this population, instead of focusing on a single chronic illness.

Our objective in this study is to use a large, de-identified private insurance claims dataset to evaluate the magnitude of association of HIV infection on developing chronic conditions while controlling for demographics, behavioral risk factors, and chronic comorbidities for patients aged 50 and older. We measure the odds ratios for acquiring chronic illness among PLWHA compared to non-PLWHA, which will further our understanding of what conditions aging PLWHA are most susceptible. In addition, we separately analyze those PLWHA diagnosed later in life, and also examine chronic disease onset after 2, 5, or 10 years from enrollment, to identify a potentially important subgroup within PLWHA which may be at particularly high risk of chronic disease.

## Methods

### Study population

This study used data from Optum’s de-identifed Clinformatics® Data Mart Database on enrollees aged 50 and above who had been followed for at least one year from Optum for the period between January 2007 and December 2016. The seven chronic disease groups of interest were diabetes, hypertension, stroke, cancers, lung diseases, cardiovascular diseases, and cognitive impairment and dementia. Cancers included prostate cancer, breast cancer, colorectal cancer, endometrial cancer, and lung cancer. Lung diseases included asthma and chronic obstructive pulmonary disease (COPD). Cardiovascular diseases included atrial fibrillation and ischemic heart diseases. Cognitive impairment and dementia included non-Alzheimer’s dementia, Alzheimer’s dementia, and cognitive impairment. These chronic diseases were identified by ICD-9 codes or ICD-10 codes (given in Supplemental Table S1).

We applied the Chronic Conditions Data Warehouse algorithm (CCW)^32^ to identify new disease onset. This included a washout period, where diagnoses for a disease within the washout period from their enrollment were not considered a “new” diagnosis. In concordance with the CCW, a 2-year washout period was applied to HIV; one-year washout period was applied to hypertension, stroke, cancers, and lung diseases; a two-year washout period was applied to diabetes and cardiovascular diseases; and a three-year washout period was applied to dementia. Using these washout periods increased the probability that the diagnosis observed was truly new and not part of an already-diagnosed condition that started prior to the patient’s enrollment.

We cannot observe the time from diagnosis for the majority of patients with HIV in the dataset – 82.5% of those with HIV were diagnosed prior to two years from enrollment, indicating that a sizable proportion were not new diagnoses. However, it is likely that time from diagnosis and exposure to antiretroviral treatment is likely to change drug toxicity and viral loads and thereby influence the severity of co-morbid chronic conditions in HIV^33–35^. We therefore perform analysis on three groups of PLWHA, which we term cohorts 1, 2, and 3 for convenience. Cohort 1 includes individuals diagnosed with HIV infection or AIDS at any time. Cohort 2 was the group of PLWHA who were diagnosed with HIV/AIDS at/before enrollment or within 2 years after enrollment. Cohort 3 was the group of PLWHA that were diagnosed with HIV/AIDS 2 years or more after their enrollment. Cohorts 2 and 3 are mutually exclusive, and together form Cohort 1. We cannot be certain of the time of HIV acquisition for individuals in any of the three cohorts, but those in Cohort 3 are diagnosed after age 50, as all individuals in our analysis are over age 50. These are therefore individuals who were living with undiagnosed HIV until later in life or acquired HIV after age 50, and we separate out these individuals for subgroup analysis as they may have different patterns of chronic disease acquisition.

### Analysis techniques and outcome measures

We report the percentage of individuals diagnosed with chronic condition for each subgroup for individuals enrolled between 2007–2016. Logistic regressions were used to estimate odds ratios of developing each chronic condition by HIV status – the odds ratio would then provide an estimate of the relative likelihood of that chronic disease for a PLWHA compared to a HIV- individual. We performed this regression for each of our three definitions of having HIV: inclusion in HIV Cohort 1 (diagnosed with HIV at any point), Cohort 2 (diagnosed with HIV/AIDS at/before enrollment or within 2 years of enrollment), or Cohort 3 (diagnosed with HIV after two years from enrollment). The dependent variable was whether the individual would develop the chronic disease by 2, 5, or 10 years from baseline (time of enrollment; separate models for each time point); only individuals without the disease at baseline were used in each regression. Other control variables included age at enrollment, sex, race/ethnicity, annual household income level at baseline, behavioral risk factors (including obesity, substance abuse, alcohol-related disease, and smoking), and the other chronic disease groups at baseline not being used as the dependent variable.

To explore model sensitivity to independent variables, we also examined different model specifications. We included demographic characteristics in our regressions because age and gender might affect the risk of acquiring chronic conditions; we also chose to include behavioral risk factors including smoking, obesity, drinking, and substance abuse as these may increase the likelihood of particular chronic illnesses^36–39^. We additionally performed sensitivity analyses on year of HIV diagnosis (as ART has changed over our analysis period) and age of HIV diagnosis for Cohort 3, where date and age of HIV diagnosis could be observed. An alpha level of 0.05 used applied to determine the statistical significance of results. Data cleaning was performed using SAS and statistical analysis were performed in Stata (StataCorp. 2017. *Stata Statistical Software: Release 15*. College Station, TX: StataCorp LLC).

### Ethics statement

All methods were carried out in accordance with relevant guidelines and regulations. On 05/19/2017, a representative of the University Park Institutional Review Board (UPIRB) at the University of Southern California determined that this study does not meet the regulatory definition of research involving human subjects. This study involved de-identified data that cannot be linked to a specific individual by the investigator(s) directly or indirectly through a coding system. In accordance with OHRP guidance on this subject (see http://www.hhs.gov/ohrp/policy/cdebiol.html), IRB approval is not required as the data cannot be tracked to a human subject. In addition, since this study did not meet regulatory definition of research involving human subjects, informed consent does not apply. The tracking number for this study was: UP-17-00343.

## Results

**Figure 1:**
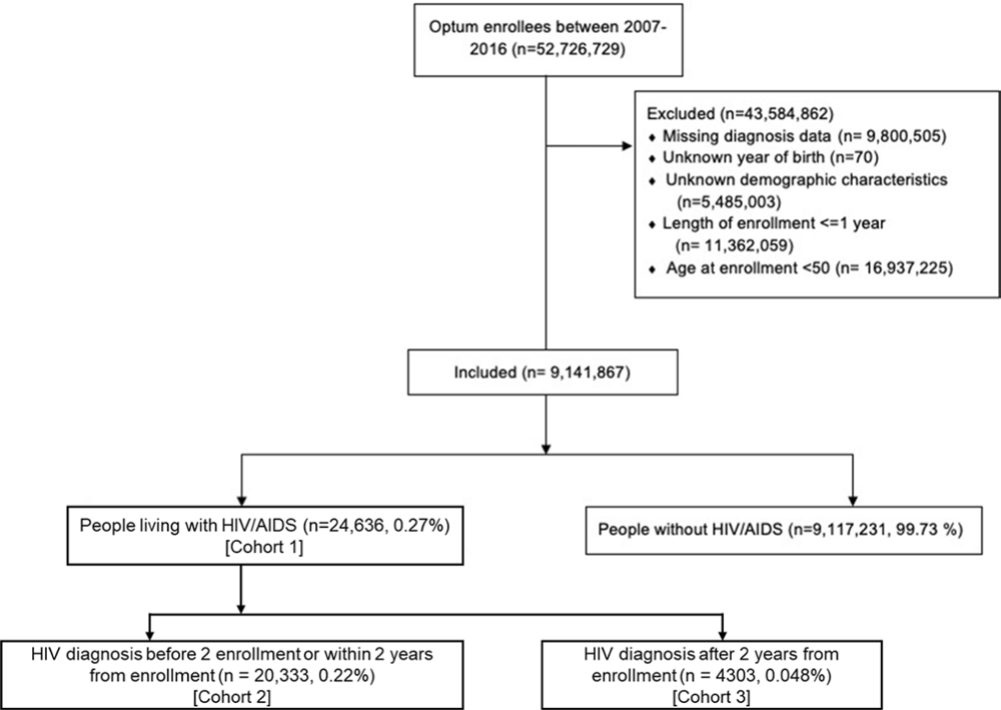
Flowchart of inclusion.

A total of 9,141,867 enrollees aged 50 and above who had been enrolled for at least 1 year were included. 24,636 (0.26%) of them were included in HIV cohort 1 (had been diagnosed with HIV/AIDS at any point) (see Fig. 1). Table 1 shows the characteristics of the entire study population at enrollment. PLWHA were more likely to be male (74.0% vs 45.8%, p-value < 0.05) and Black (28.7% vs 10.4%, p-value < 0.05). PLWHA were more likely to earn less compared to people without HIV/AIDS (45.6% had annual household income less than $50k, compared to 33.2% among HIV- individuals). PLWHA were also more likely to be diagnosed with alcohol-related disease (2.0% vs 0.7%) and substance abuse (0.6% vs 0.1%). Smoking was more common among PLWHA (21.5% vs 11%). Individuals diagnosed with HIV after 2 years from enrollment (Cohort 3) were also more likely to be older during the analysis period, female, obese, abuse alcohol, and have higher prevalence of diabetes, hypertension, cardiovascular disease, and cognitive impairment at enrollment compared to those diagnosed before or soon after enrollment (Cohort 2), as shown in Table 1. Summary statistics on chronic disease incidence by cohort is given in Supplemental Table S2.

**Table 1:** Summary Statistics. Baseline Characteristics at Enrollment.

	HIV− Throughout Enrollment	HIV+ Cohort 1^^^	HIV+ Cohort 2^^^	HIV+ Cohort 3^^^
Mean age	64.1	59.2*	58.6	62.1**
Male (%)	45.8	74.0*	77.0	59.6**
Female (%)	54.2	26.0*	23.0	40.4**
White (%)	76.5	56.2*	56.2	56.2
Black (%)	10.4	28.7*	29.3	25.4**
Hispanic (%)	9.60	13.3*	12.9	15.6**
Education level < High School (%)	0.70	0.90*	0.90	1.20
Annual Household Income < 50 K (%)	33.2	45.6*	46.4	42.2**
Obesity (%)	17.6	17.2*	15.1	27.5**
Alcohol-related disease (%)	0.70	2.00*	1.80	3.20**
Substance abuse (%)	0.10	0.60*	0.60	0.40
Smoking (%)	11.7	21.5*	21.3	22.4
Diabetes (%)	24.2	28.8*	28.0	32.9**
Hypertension (%)	46.4	46.9*	46.4	49.2**
Stroke (%)	2.40	3.20*	3.20	3.30
Cancer (%)	5.70	4.80*	4.70	5.10
Lung disease (%)	11.3	14.3*	14.2	14.7
Cardiovascular disease (%)	21.2	22.3*	21.6	25.3**
Cognitive impairment and dementia (%)	8.00	9.20*	8.60	11.8**

^^^HIV Cohort 1 (diagnosed with HIV at any point), Cohort 2 (diagnosed with HIV prior to two years from enrollment), or Cohort 3 (diagnosed with HIV after two years from enrollment). *Differences between HIV- and Cohort 1 values are statistically significantly different for every characteristic, with p-values < 0.05.

**Differences between Cohort 2 and Cohort 3 values are statistically significantly different, with p-values < 0.05.

The average length of enrollment in the insurance plan was 4.40 years for PLWHA and 4.54 years for people without HIV/AIDS. While this difference of 1.68 months was statistically significant (p < 0.05), it is likely too small to be clinically meaningful. PLWHA tended to enroll at younger ages compared to people without HIV/AIDS. The average age at enrollment was 59.2 years old (95% CI: 59.1–59.3) for cohort 1 and 64.1 years old (95% CI: 64.1–64.2) for people without HIV/AIDS. Moreover, 75% of PLWHA were younger than 65 years old at enrollment (78% of cohort 2 and 60% of cohort 3), providing a sizable proportion of relatively younger patients for our study, even in cohort 3.

In general, PLWHA (in any HIV cohort) were more likely to have chronic conditions at the time they were enrolled. PLWHA had higher prevalence of diabetes (28.8% vs 24.2%), hypertension (46.9% vs 46.4%), stroke (3.3% vs 2.4%), lung diseases (14.3% vs 11.3%), cardiovascular diseases (22.3% vs 21.2%), and dementia (9.2% vs 8.0%) at enrollment. People living without HIV/AIDS appeared to have higher prevalence of cancer than PLWHA (4.8% vs 5.7%) at enrollment. All differences were statistically significant at an alpha level of 0.05, but not all differences may be clinically meaningful.

We ran multivariable logistic regressions for the onset of each chronic condition within two years of enrollment. Results for cognitive impairment/dementia onset when HIV is defined as inclusion in HIV cohort 1 is shown in Table 2 as an example; the outcomes for the remaining diseases are shown in the Supplemental Table S3. Relative risks (instead of odds ratios) are provided in Supplemental Tables S4–S6 (with description of how this analysis was performed). The outcomes when the model specification is varied are shown in Supplemental Tables S7–S13). Table 3 provides a summary of the odds ratios of developing the indicated chronic illness for an individual in HIV Cohort 1 compared to someone without HIV, controlling for all the covariates listed in Table 2.

**Table 2:** Logistic Regression Outcomes.

	Diabetes	Hypertension	Stroke	Cancer	Lung Disease	Cardiovascular Disease	Cog. Impairment/Dementia
HIV/AIDS status at enrollment	1.30	1.13	1.28	1.44	1.27	1.30	1.66
	(1.19–1.42)	(1.06–1.20)	(1.16–1.40)	(1.31–1.58)	(1.19–1.35)	(1.20–1.40)	(1.48–1.86)
Observations	4829266	3381256	6215432	5981854	5658282	5027839	5907482

2-Year Odds Ratios Compared with HIV- enrollees. *The HIV variable is defined by Cohort 1, ever diagnosed with HIV at any point. Control variables include demographic variables, comorbidities, and behavioral factors.

*Robust 95% CI in parentheses. All values significant at the 5% level unless in italics. Significance of the HIV odds ratio is robust to model specifications (see Supplemental Table S3).

**Table 3:** Odds ratios for HIV in logistic regressions for Cohort 1 (ever diagnosed with HIV), using different intervals for chronic disease onset.

Interval	Diabetes	Hypertension	Stroke	Cancer	Lung Disease	Cardiovascular Disease	Cognitive Impairment & Dementia
2-year	1.30	1.13	1.28	1.44	1.26	1.30	1.66
	(1.19–1.42)	(1.06–1.20)	(1.16–1.40)	(1.31–1.58)	(1.19–1.34)	(1.20–1.40)	(1.48–1.86)
5-year	1.44	1.22	1.43	1.44	1.25	1.38	1.56
	(1.33–1.57)	(1.13–1.31)	(1.30–1.57)	(1.31–1.58)	(1.17–1.34)	(1.28–1.49)	(1.40–1.72)
10-year	1.45	1.36	1.45	1.37	1.33	1.37	1.73
	(1.24–1.70)	(1.17–1.58)	(1.22–1.73)	(1.15–1.64)	(1.16–1.52)	(1.19–1.58)	(1.44–2.08)

95% CI in Parentheses. All values statistically significantly different from 1 at the 5% level unless in italics.

We find that the odds ratios for chronic illness for PLWHA, compared to HIV- individuals, were statistically significant at the 5% level and were greater than 1. This indicates that the risk for the onset of these chronic conditions were higher among this PLWHA group, even controlling for comorbidities, demographics, and behavioral differences. To examine the robustness of these results, we reran the analysis where the dependent variable was changed to the onset of each of these chronic conditions within 5 and 10 years from enrollment; the odds ratios remained very similar in magnitude and remained statistically significant, indicating that disease onset risk may be relatively constant over time (see Table 3). The odds ratios across all chronic conditions were between 1.1 and 1.8, with the highest odds ratio for cognitive impairment and dementia. Given the large sample size in this study, even small differences would be statistically significant, but in general, these odds ratios are similar in size to many of those seen in our outcomes for behavioral risk factors such as smoking, alcohol, or substance use (see tables in Supplemental Information), indicating that the risk due to HIV may be comparable in magnitude to these established risk factors for chronic illness.

Table 4 presents the corresponding results when we limit our HIV definition to Cohort 2, PLWHA who were diagnosed with HIV/AIDS at/before two years after enrollment (upper half of table), and Cohort 3, PLWHA who were diagnosed with HIV/AIDS two years or more after enrollment (lower half of table), controlling for all the demographic, disease, and behavioral variables listed above. Here, we find that the odds ratios of developing the indicated chronic illness for Cohort 2 compared to HIV- individuals was only sometimes statistically significant, depending on the interval on which onset was defined.

**Table 4:** Odds Ratios for HIV in logistic regressions for Cohort 2 & 3*, using different intervals for chronic disease onset.

Interval	Diabetes	Hypertension	Stroke	Cancer	Lung Disease	Cardiovascular Disease	Cognitive Impairment & Dementia
**Cohort 2**							
2-year	0.88	0.90	*1.02*	1.17	*1.04*	*0.94*	1.18
	(0.78–0.99)	(0.84–0.97)	(0.90–1.15)	(1.04–1.31)	(0.97–1.13)	(0.85–1.04)	(1.01–1.38)
5-year	*1.09*	*1.07*	1.23	1.34	*1.05*	1.11	1.33
	(0.97–1.22)	(0.98–1.17)	(1.08–1.40)	(1.19–1.51)	(0.96–1.15)	(1.00–1.22)	(1.15–1.54)
10-year	*1.18*	1.23	1.40	1.31	*1.14*	*1.19*	1.75
	(0.95–1.47)	(1.00–1.49)	(1.09–1.81)	(1.03–1.68)	(0.95–1.37)	(0.97–1.47)	(1.34–2.29)
**Cohort 3**							
2-year	2.92	2.10	2.02	2.30	2.04	2.51	2.97
	(2.56–3.34)	(1.88–2.34)	(1.73–2.37)	(1.98–2.68)	(1.83–2.27)	(2.22–2.84)	(2.50–3.51)
5-year	2.17	1.55	1.73	1.59	1.63	1.92	1.87
	(1.92–2.46)	(1.37–1.75)	(1.50–1.99)	(1.38–1.84)	(1.46–1.81)	(1.71–2.15)	(1.61–2.17)
10-year	1.84	1.57	1.50	1.44	1.58	1.58	1.71
	(1.47–2.30)	(1.24–1.98)	(1.17–1.92)	(1.11–1.86)	(1.30–1.92)	(1.29–1.95)	(1.34–2.20)

95% CI in Parentheses. All values statistically significantly different from 1 at the 5% level unless in italics.

*Cohort 2 (diagnosed with HIV prior to two years from enrollment); Cohort 3 (diagnosed with HIV after two years from enrollment). Comparison group: HIV- individuals.

Notably, the odds ratio of chronic disease onset was higher when HIV status was limited to Cohort 3. The odds ratios were all statistically significant, and many were above 2 (see Table 4). While the odds ratios tended to decrease with longer onset intervals, these values are generally larger than those found when HIV was defined by Cohort 1 or Cohort 2, meaning that individuals diagnosed with HIV after age 50 are generally at higher risk of chronic disease onset compared with HIV- individuals, and even PLWHA who were diagnosed before enrollment. Results from the additional sensitivity analyses on year of HIV diagnosis and age of HIV diagnosis for Cohort 3 are shown in Supplemental Tables S14–S17 and show similar results.

## Discussion

This study analyzed the risk of developing seven chronic conditions including diabetes, hypertension, stroke, cancers, lung diseases, cardiovascular diseases, and dementia using a large de-identified private insurance claims database for people who were 50 years of age or older, stratified by HIV status. We found that PLWHA were more likely to have chronic conditions even controlling for demographic characteristics, behavioral risk factors, and other chronic comorbidities. Our findings were robust to different follow-up periods of analysis and under different model specifications.

HIV infection had an even greater association on the development of these chronic conditions among those who were diagnosed with HIV/AIDS later, as evidenced by our results for individuals diagnosed with HIV two years after enrollment (Cohort 3). This could be because these individuals had undiagnosed HIV/AIDS for a longer period (as they were diagnosed after age 50), or had some other unobserved characteristics correlated to acquiring HIV later in life. This finding spurs additional future research on the relationship between HIV diagnosis age and long-term health outcomes.

We must acknowledge several limitations of this study. To perform our analysis, we used private insurance claims data, which may not be nationally representative. For instance, individuals in our analysis tend to have higher than average socioeconomic status, as measured through income (see Table 1). However, this would likely mean that this population is, on average, healthier than the national average, leading to conservative estimates of the elevated risk of chronic disease in this population. This population may not be generalizable to the national US population on other characteristics as well, some of which may not be observable. We chose to limit our analysis to only those aged 50 and older, as these individuals may be at most risk for chronic conditions which tend to be exacerbated by age. Note that these results are also not generalizable to the younger PLWHA population, as those who died younger than 50 could be sicker; our results pertain only to the aging PLWHA population. In general, we were unable to control for competing mortality risk, and this could have potentially led to some counter-intuitive findings (such as higher prevalence of cancer in the HIV- group at enrollment).

We also cannot control for the duration an individual lived with HIV, nor what fraction of that time was spent on ART, adherence levels on ART, nor what type of ART was used prior to enrollment in this dataset. Many of the PLWHA in our analysis were diagnosed with HIV prior to enrollment (82.5%), and we cannot observe the date of diagnosis nor their treatment. Prior studies have identified the duration with HIV and on ART as potentially important in the acquisition of chronic illness among PLWHA. However, even limiting the PLWHA population to those who had been previously diagnosed with HIV (Cohort 2), we found evidence of elevated chronic disease risk. Our data was similarly lacking for behavioral factors; we had to rely on coded diagnoses of smoking and substance abuse – which are likely to only able to identify extreme cases of these risk factors. We additionally note that our analysis does not explicitly account for the timing of diagnosis of HIV relative to chronic illness (instead separating out diagnosis of HIV either before two years from enrollment or afterwards), and we cannot conclude that HIV causes elevated risk for chronic illnesses, merely that the two are associated.

Despite these limitations, we believe that our findings shed light on the important issue of chronic disease among aging PLWHA. Even after controlling for co-morbidities and behavioral effects, we found that PLWHA prior to age 65 have elevated risk for a variety of chronic illnesses. While not nationally generalizable, this study includes a much larger PLWHA sample size than in many prior studies, and comparable in population size with the largest studies on this topic – many studies use hundreds, or even thousands, of patients, while those with over 24,000 PLWHA are rare^16,33^. We also control, at least in a limited way, for multiple comorbidities and behavior risk factors, which are known to affect the acquisition of further illnesses. We are also able to shed light on the pre-65 PLWHA age group, which may have had less emphasis in the US in prior work.

Critically, the results of this analysis suggest that while PLWHA are at higher risk in general for acquiring chronic illness, those PLWHA who are diagnosed after age 50 are at particular risk. The differences in magnitude in the odds ratios between Cohort 2 and Cohort 3 were stark, with odds ratios for chronic illness often above 2 for our Cohort 3 results. In our sample, this population tended to be older and more, female, overweight, and abuse alcohol, but these results persisted even after controlling for these characteristics. This could be due to acquisition of HIV later in life–other studies have documented that women were more likely to be older than diagnosis than men^40^ – or through delayed diagnosis, which could arise for a variety of reasons. These include lack of access to care or HIV stigma and fear of testing^41^. We acknowledge that PLWHA in Cohort 3 tend to be older, and therefore subject to bias – they only enter Cohort 3 if they are still alive to be diagnosed two years after enrollment in the dataset.

This study contributes towards our understanding of chronic illness among PLWHA prior to age 65. With an aging PLWHA population, both medical practitioners and health policymakers must understand the risks this growing vulnerable population faces as they are exposed to the natural aging process in addition to unique risks due to HIV and HIV treatment. Quantification of the magnitude of chronic disease risk will enable more tailored preventative and screening programs for this population. In future work, we hope to explore more finely age-stratified chronic disease risk in this population, although this dataset was not large enough to support finely stratified age-group analysis, particularly given the number of control variables needed. This study’s results also indicate that further work is needed to understand the role of HIV diagnosis age in relationship to chronic disease onset. We hope that the findings in this study will contribute to a growing body of literature which will help our healthcare system prepare for a rapidly increasing population of aging PLWHA.

## Supplementary information

Supplemental Information File

## Data Availability

The data that support the findings of this study, Optum’s de-identifed Clinformatics® Data Mart Database, were used under license and are not publicly available. The data are available from Optum for a fee.
