# Supplementary material for: Chronic Disease Onset Among People Living with HIV and AIDS in a Large Private Insurance Claims Dataset
Source: Sci Rep. 2019 Dec 6;9:18514. doi: 10.1038/s41598-019-54969-3 (PMC6897968; doi:10.1038/s41598-019-54969-3)
Supplement: Supplementary file 1 — Supplemental Information File [file 41598_2019_54969_MOESM1_ESM.pdf]

Supplemental Information for:

Chronic Disease Onset Among People Living with HIV and AIDS in a Large Private Insurance Claims Dataset

Hsin-Yun Yang, Matthew Beymer, Sze-chuan Suen

Table S1: ICD-9 and ICD-10 codes used in analysis		
**Condition**	**ICD-9 Codes**	**ICD-10 Codes**
HIV/AIDS	042, 042.0, 042.1, 042.2, 042.9, 043, 043.1, 043.2, 043.3, 043.9, 044, 044.0, 044.9, 079.53, V08	B20-B24, B97.35, Z21
Diabetes	249.00, 249.01, 249.10, 249.11, 249.20, 249.21, 249.30, 249.31, 249.40, 249.41, 249.50, 249.51, 249.60, 249.61, 249.70, 249.71, 249.80, 249.81, 249.90, 249.91, 250.00, 250.01, 250.02, 250.03, 250.10, 250.11, 250.12, 250.13, 250.20, 250.21, 250.22, 250.23, 250.30, 250.31, 250.32, 250.33, 250.40, 250.41, 250.42, 250.43, 250.50, 250.51, 250.52, 250.53, 250.60, 250.61, 250.62, 250.63, 250.70, 250.71, 250.72, 250.73, 250.80, 250.81, 250.82, 250.83, 250.90, 250.91, 250.92, 250.93, 357.2, 362.01, 362.02, 362.03, 362.04, 362.05, 362.06, 366.41	E08.00, E08.01, E08.10, E08.11, E08.21, E08.22, E08.29, E08.311, E08.319, E08.321, E08.329, E08.331, E08.339, E08.341, E08.349, E08.351, E08.359, E08.36, E08.39, E08.40, E08.41, E08.42, E08.43, E08.44, E08.49, E08.51, E08.52, E08.59, E08.610, E08.618, E08.620, E08.621, E08.622, E08.628, E08.630, E08.638, E08.641, E08.649, E08.65, E08.69, E08.8, E08.9, E09.00, E09.01, E09.10, E09.11, E09.21, E09.22, E09.29, E09.311, E09.319, E09.321, E09.329, E09.331, E09.339, E09.341, E09.349, E09.351, E09.359, E09.36, E09.39, E09.40, E09.41, E09.42, E09.43, E09.44, E09.49, E09.51, E09.52, E09.59, E09.610, E09.618, E09.620, E09.621, E09.622, E09.628, E09.630, E09.638, E09.641, E09.649, E09.65, E09.69, E09.8, E09.9, E10.10, E10.11, E10.21, E10.22, E10.29, E10.311, E10.319, E10.321, E10.329, E10.331, E10.339, E10.341, E10.349, E10.351, E10.359, E10.36, E10.39, E10.40, E10.41, E10.42, E10.43, E10.44, E10.49, E10.51, E10.52, E10.59, E10.610, E10.618, E10.620, E10.621, E10.622, E10.628, E10.630, E10.638, E10.641, E10.649, E10.65, E10.69, E10.8, E10.9, E11.00, E11.01, E11.21, E11.22, E11.29, E11.311, E11.319, E11.321, E11.329, E11.331, E11.339, E11.341, E11.349, E11.351, E11.359, E11.36, E11.39, E11.40, E11.41, E11.42, E11.43, E11.44, E11.49, E11.51, E11.52, E11.59, E11.610, E11.618, E11.620, E11.621, E11.622, E11.628, E11.630, E11.638, E11.641, E11.649, E11.65, E11.69, E11.8, E11.9, E13.00, E13.01, E13.10, E13.11, E13.21, E13.22, E13.29, E13.311, E13.319, E13.321, E13.329, E13.331, E13.339, E13.341, E13.349, E13.351, E13.359, E13.36, E13.39, E13.40, E13.41, E13.42, E13.43, E13.44, E13.49, E13.51, E13.52, E13.59, E13.610, E13.618, E13.620, E13.621, E13.622, E13.628, E13.630, E13.638, E13.641, E13.649, E13.65, E13.69, E13.8, E13.9
Hypertension	362.11, 401.0, 401.1, 401.9, 402.00, 402.01, 402.10, 402.11, 402.90, 402.91, 403.00, 403.01, 403.10, 403.11, 403.90, 403.91, 404.00, 404.01, 404.02, 404.03, 404.10, 404.11, 404.12, 404.13, 404.90, 404.91, 404.92, 404.93, 405.01, 405.09, 405.11, 405.19, 405.91, 405.99, 437.2	H35.031, H35.032, H35.033, H35.039, I10, I11.0, I11.9, I12.0, I12.9, I13.0, I13.10, I13.11, I13.2, I15.0, I15.1, I15.2, I15.8, I15.9, I67.4, N26.2

Stroke	430, 431, 433.01, 433.11, 433.21, 433.31, 433.81, 433.91, 434.00, 434.01, 434.10, 434.11, 434.90, 434.91, 435.0, 435.1, 435.3, 435.8, 435.9, 436, 997.02	G45.0, G45.1, G45.2, G45.8, G45.9, G46.0, G46.1, G46.2, G97.31, G97.32, I60.00, I60.01, I60.02, I60.10, I60.11, I60.12, I60.20, I60.21, I60.22, I60.30, I60.31, I60.32, I60.4, I60.50, I60.51, I60.52, I60.6, I60.7, I60.8, I60.9, I61.0, I61.1, I61.2, I61.3, I61.4, I61.5, I61.6, I61.8, I61.9, I63.00, I63.02, I63.011, I63.012, I63.019, I63.031, I63.032, I63.039, I63.09, I63.10, I63.111, I63.112, I63.119, I63.12, I63.131, I63.132, I63.139, I63.19, I63.20, I63.211, I63.212, I63.219, I63.22, I63.231, I63.232, I63.239, I63.29, I63.30, I63.311, I63.312, I63.319, I63.321, I63.322, I63.329, I63.331, I63.332, I63.339, I63.341, I63.342, I63.349, I63.39, I63.40, I63.411, I63.412, I63.419, I63.421, I63.422, I63.429, I63.431, I63.432, I63.439, I63.441, I63.442, I63.449, I63.49, I63.50, I63.511, I63.512, I63.519, I63.521, I63.522, I63.529, I63.531, I63.532, I63.539, I63.541, I63.542, I63.549, I63.59, I63.6, I63.8, I63.9, I66.01, I66.02, I66.03, I66.09, I66.11, I66.12, I66.13, I66.19, I66.21, I66.22, I66.23, I66.29, I66.3, I66.8, I66.9, I67.841, I67.848, I67.89, I97.810, I97.811, I97.820, I97.821
Cancers	185, 233.4, V10.46, 162.2, 162.3, 162.4, 162.5, 162.8, 162.9, 231.2, V10.11,174.0, 174.1, 174.2, 174.3, 174.4, 174.5, 174.6, 174.8, 174.9, 175.0, 175.9, 233.0, V10.3, 182.0, 233.2, V10.42, 153.0, 153.1, 153.2, 153.3, 153.4, 153.5, 153.6, 153.7, 153.8, 153.9,154.0,154.1, 230.3, 230.4, V10.05, V10.06	C61, D07.5, Z85.46, C34.00, C34.01, C34.02, C34.10, C34.11, C34.12, C34.2, C34.30, C34.31, C34.32, C34.80, C34.81, C34.82, C34.90, C34.91, C34.92, D02.20, D02.21, D02.22, Z85.118, C50.011, C50.012, C50.019, C50.021, C50.022, C50.029, C50.111, C50.112, C50.119, C50.121, C50.122, C50.129, C50.211, C50.212, C50.219, C50.221, C50.222, C50.229, C50.311, C50.312, C50.319, C50.321, C50.322, C50.329, C50.411, C50.412, C50.419, C50.421, C50.422, C50.429, C50.511, C50.512, C50.519, C50.521, C50.522, C50.529, C50.611, C50.612, C50.619, C50.621, C50.622, C50.629, C50.811, C50.812, C50.819, C50.821, C50.822, C50.829, C50.911, C50.912, C50.919, C50.921, C50.922, C50.929, D05.00, D05.01, C18.0, C18.1, C18.2, C18.3, C18.4, C18.5, C18.6, C18.7, C18.8, C18.9, C19, C20, D01.0, D01.1, D01.2, Z85.038, Z85.048 D05.02, D05.10, D05.11, D05.12, D05.80, D05.81, D05.82, D05.90, D05.91, D05.92, Z85.3, C54.1, C54.2, C54.3, C54.9, D07.0, Z85.42,
Lung Diseases	490, 491.0, 491.1, 491.8, 491.9, 492.0, 492.8, 491.20, 491.21, 491.22, 494.0, 494.1, 496, 493.00, 493.01, 493.02, 493.10, 493.11, 493.12, 493.20, 493.21, 493.22, 493.81, 493.82, 493.90, 493.91, 493.92,	J40, J41.0, J41.1, J41.8, J42, J43.0, J43.1, J43.2, J43.8, J43.9, J44.0, J44.1, J44.9, J47.0, J47.1, J47.9, J45.20, J45.21, J45.22, J45.30, J45.31, J45.32, J45.40, J45.41, J45.42, J45.50, J45.51, J45.52, J45.901, J45.902, J45.909, J45.990, J45.991, J45.998
Cardiovascular Diseases	427.31, 410.00, 410.01, 410.02, 410.10, 410.11, 410.12, 410.20, 410.21, 410.22, 410.30, 410.31, 410.32, 410.40, 410.41, 410.42, 410.50, 410.51, 410.52, 410.60, 410.61, 410.62, 410.70, 410.71, 410.72, 410.80, 410.81, 410.82, 410.90, 410.91, 410.92, 411.0, 411.1, 411.81, 411.89, 412, 413.0, 413.1, 413.9, 414.00, 414.01, 414.02, 414.03, 414.04, 414.05, 414.06, 414.07, 414.12, 414.2, 414.3, 414.4, 414.8, 414.9	I48.0, I48.2, I48.91, I20.0, I20.1, I20.8, I20.9, I21.01, I21.02, I21.09, I21.11, I21.19, I21.21, I21.29, I21.3, I21.4, I22.0, I22.1, I22.2, I22.8, I22.9, I24.0, I24.1, I24.8, I24.9, I25.10, I25.110, I25.111, I25.118, I25.119, I25.2, I25.42, I25.5, I25.6, I25.700, I25.701, I25.708, I25.709, I25.710, I25.711, I25.718, I25.719, I25.720, I25.721, I25.728, I25.729, I25.730, I25.731, I25.738, I25.739, I25.750, I25.751, I25.758, I25.759, I25.760, I25.761, I25.768, I25.769, I25.790, I25.791, I25.798, I25.799, I25.810, I25.811, I25.812, I25.82, I25.83, I25.84, I25.89, I25.9
Cognitive Impairment & Dementia	331.82, 331.89, 331.9, 290.8, 290.9, 294.9, 780.93, 784.3, 784.69, 331.83, 331.0, 331.11, 331.19, 331.2, 331.7, 797, 290.0, 290.10, 290.11, 290.12, "29013", 290.20, 290.21, 290.3, 290.40, 290.41, 290.42, 290.43, 294.0, 294.10, 294.11, 294.20, 294,21, 294.8	F01.50, F01.51, F02.80, F02.81, F03.90, F03.91, G13.8, F06.1, F06.8, G30.0, G30.1, G30.8, G30.9, G31.1, G31.2, G31.01, G31.09, R41.81, F04, F05, G94, R54, G31.83, G31.89, G31.9, F03.90, F03.90, R41.2, R41.3, R47.01, R48.1, R48.2, R48.8, G31.84

Obesity	278.00	E66.0, E66.01, E66.09. E66.1 E66.2 E66.3 E66.8 E66.9
Alcohol use (abuse/alcohol-related disorder)	305.0 303.0 303.9, 291, 357.5, 425.5, 535.3, 571.0, 571.1, 571.2, 571.3, 655.4, 760.71	F10.0 F10.1 F10.2 F10.9, G62.1, G31.2, G72.1, I42.6, K29.2, K70, Q86, P04.3, O35.4, K86.0
Substance abuse	305.9	F19.10
Smoking	305.1	F17

Incidence Summary Statistics

Table S2. Incidence: New cases per 100 individuals at risk in each cohort*

	HIV-	HIV+ Cohort 1	HIV+ Cohort 2	HIV+ Cohort 3
Diabetes	7.93	10.96	7.25	29.74
Hypertension	40.69	43.88	38.82	69.01
Stroke	6.22	7.3	5.43	16.13
Cancer	6.05	6.79	5.5	12.9
Lung Diseases	16.49	20.15	16.63	36.88
Cardiovascular Diseases	11.61	13.41	9.06	34.95
Dementia	4.66	4.62	2.69	14.09

*HIV Cohort 1 (diagnosed with HIV at any point), Cohort 2 (diagnosed with HIV prior to two years from enrollment), or Cohort 3 (diagnosed with HIV after two years from enrollment)

Outcomes for Control Variables in Logistic Regression (Continuation of Table 2)

Table S3 (Continuation of Table 2 from main text): Logistic Regression Outcomes. 2-Year Odds Ratios Compared with HIV-enrollees.* The HIV variable is defined by Cohort 1, ever diagnosed with HIV

	Diabetes	Hyper-tension	Stroke	Cancer	Lung Disease	Cardiovascular Disease	Cog. Impairment/ Dementia
Age at enrollment	1.02 (1.01 - 1.01)	1.03 (1.03 - 1.03)	1.05 (1.04 - 1.04)	1.05 (1.04 - 1.04)	1.02 (1.02 - 1.02)	1.06 (1.05 - 1.05)	1.10 (1.09 - 1.09)
Male	1.20 (1.19 - 1.21)	1.21 (1.20 - 1.22)	1.04 (1.02 - 1.05)	1.24 (1.22 - 1.25)	0.91 (0.90 - 0.91)	1.32 (1.31 - 1.33)	0.91 (0.89 - 0.92)
Black	1.28 (1.26 - 1.30)	1.13 (1.11 - 1.14)	1.07 (1.05 - 1.09)	1.04 (1.02 - 1.06)	0.87 (0.86 - 0.88)	0.96 (0.95 - 0.97)	0.96 (0.93 - 0.97)
Annual household income < 50K	1.23 (1.21 - 1.23)	1.16 (1.14 - 1.16)	1.18 (1.17 - 1.19)	1.06 (1.04 - 1.06)	1.21 (1.20 - 1.22)	1.18 (1.16 - 1.18)	1.22 (1.20 - 1.23)
Diabetes status at enrollment	1.13 (1.11 - 1.13)	0.95 (0.94 - 0.96)	1.22 (1.20 - 1.22)	*0.99* (0.98 - 1.00)	1.05 (1.03 - 1.05)	1.22 (1.20 - 1.23)	1.09 (1.07 - 1.10)
Stroke status at enrollment	*0.97* (0.93 - 1.00)	*1.01* (0.96 - 1.04)	*1.00* (0.98 - 1.00)	0.79 (0.77 - 0.79)	0.88 (0.87 - 0.88)	1.01 (1.00 - 1.02)	0.81 (0.79 - 0.82)
Cancer status at enrollment	0.86 (0.84 - 0.87)	0.87 (0.85 - 0.88)	0.93 (0.90 - 0.94)	0.85 (0.82 - 0.88)	0.93 (0.90 - 0.95)	1.28 (1.24 - 1.32)	1.41 (1.36 - 1.45)
Lung disease status at enrollment	1.02 (1.00 - 1.04)	0.97 (0.96 - 0.98)	1.01 (1.00 - 1.03)	1.12 (1.10 - 1.13)	*1.01* (0.99 - 1.02)	0.91 (0.89 - 0.93)	0.92 (0.89 - 0.93)
Cardiovascular disease status at enrollment	1.22 (1.20 - 1.24)	0.82 (0.81 - 0.83)	1.57 (1.54 - 1.58)	0.99 (0.97 - 0.99)	1.25 (1.23 - 1.25)	1.17 (1.15 - 1.19)	*1.01* (0.99 - 1.02)
Cog. impairment and dementia status at enrollment	*1.00* (0.98 - 1.02)	1.15 (1.13 - 1.17)	3.51 (3.46 - 3.55)	1.07 (1.05 - 1.09)	1.33 (1.30 - 1.34)	1.14 (1.12 - 1.15)	1.25 (1.23 - 1.26)
Obesity status at enrollment	2.50 (2.47 - 2.53)	1.70 (1.68 - 1.70)	1.33 (1.31 - 1.34)	1.35 (1.32 - 1.36)	1.56 (1.54 - 1.57)	1.98 (1.95 - 1.99)	1.42 (1.40 - 1.44)
Alcohol-related disease status at enrollment	1.83 (1.74 - 1.91)	1.61 (1.55 - 1.66)	1.58 (1.51 - 1.65)	1.32 (1.25 - 1.39)	1.70 (1.64 - 1.75)	2.05 (1.97 - 2.12)	2.90 (2.77 - 3.04)
Substance abuse status at enrollment	*0.98* (0.80 - 1.19)	1.27 (1.10 - 1.45)	1.57 (1.33 - 1.85)	0.73 (0.56 - 0.93)	*0.95* (0.82 - 1.09)	1.21 (1.02 - 1.42)	2.22 (1.81 - 2.70)
Smoking status at enrollment	1.39 (1.36 - 1.40)	1.44 (1.42 - 1.45)	1.77 (1.74 - 1.79)	1.78 (1.75 - 1.80)	2.64 (2.62 - 2.66)	2.02 (2.00 - 2.04)	1.72 (1.69 - 1.74)
Observations	4829266	3381256	6215432	5981854	5658282	5027839	5907482

*Robust 95% CI in parentheses. All values significant at the 5% level unless in italics. Significance of the HIV odds ratio is robust to model specifications

Relative Risks

It may be useful to additionally see report the relative risks associated with HIV (instead of odds ratios). These results, for each of the cohorts, are presented here. HIV Cohort 1 (diagnosed with HIV at any point), Cohort 2 (diagnosed with HIV prior to two years from enrollment), or Cohort 3 (diagnosed with HIV after two years from enrollment). Relative risks are estimated by using the Stata post-estimation command nlcom after running the logistic regression using the logit command and margins command. After running the margins command, the post estimation command nlcom estimates the ratio of marginal risks, i.e. the ratio of the estimate for PLWHA versus the estimate for people without HIV/AIDS.

Table S4. HIV Relative Risks for HIV Cohort 1 (diagnosed with HIV at any point) Compared with HIV- Enrollees

Interval	Diabetes	Hypertension	Stroke	Cancer	Lung diseases	Cardiovascular diseases	Dementia
2-year	1.36	1.10	1.48	1.32	1.26	1.35	1.57
	(1.29 - 1.42)	(1.07 - 1.12)	(1.40 - 1.56)	(1.24 - 1.39)	(1.22 - 1.31)	(1.29 - 1.40)	(1.46 - 1.68)
5-year	1.44	1.14	1.46	1.34	1.26	1.39	1.55
	(1.36 - 1.52)	(1.11 - 1.16)	(1.36 - 1.56)	(1.25 - 1.43)	(1.21 - 1.31)	(1.32 - 1.45)	(1.43 - 1.68)
10-year	1.37	1.12	1.41	1.27	1.29	1.33	1.60
	(1.23 - 1.51)	(1.08 - 1.15)	(1.21 - 1.60)	(1.09 - 1.45)	(1.20 - 1.38)	(1.23 - 1.44)	(1.38 - 1.83)

95% CI in parentheses. All relative risks statistically significantly different from 1 at the 5% level.

Table S5. HIV Relative Risks for HIV Cohort 2 (diagnosed with HIV prior to two years from enrollment) Compared with HIV-Enrollees

Interval	Diabetes	Hypertension	Stroke	Cancer	Lung diseases	Cardiovascular diseases	Dementia
2-year	0.94	0.99	1.14	1.13	1.07	0.97	1.07
	(0.88 - 1.01)	(0.97 - 1.02)	(1.06 - 1.23)	(1.05 - 1.20)	(1.02 - 1.11)	(0.92 - 1.03)	(0.97 - 1.18)
5-year	1.44	1.14	1.46	1.34	1.26	1.39	1.55
	(1.36 - 1.52)	(1.11 - 1.16)	(1.36 - 1.56)	(1.25 - 1.43)	(1.21 - 1.31)	(1.32 - 1.45)	(1.43 - 1.68)
10-year	1.37	1.12	1.41	1.27	1.29	1.33	1.60
	(1.23 - 1.51)	(1.08 - 1.15)	(1.21 - 1.60)	(1.09 - 1.45)	(1.20 - 1.38)	(1.23 - 1.44)	(1.38 - 1.83)

95% CI in parentheses. All relative risks statistically significantly different from 1 at the 5% level.

Table S6. HIV Relative Risks for HIV Cohort 3 (diagnosed with HIV after two years from enrollment) Compared with HIV- Enrollees

Interval	Diabetes	Hypertension	Stroke	Cancer	Lung diseases	Cardiovascular diseases	Dementia
2-year	2.96	1.52	2.47	1.92	1.96	2.69	2.99
	(2.75 - 3.16)	(1.47 - 1.57)	(2.27 - 2.68)	(1.74 - 2.09)	(1.86 - 2.06)	(2.53 - 2.85)	(2.69 - 3.29)
5-year	1.44	1.14	1.46	1.34	1.26	1.39	1.55
	(1.36 - 1.52)	(1.11 - 1.16)	(1.36 - 1.56)	(1.25 - 1.43)	(1.21 - 1.31)	(1.32 - 1.45)	(1.43 - 1.68)
10-year	1.37	1.12	1.41	1.27	1.29	1.33	1.60
	(1.23 - 1.51)	(1.08 - 1.15)	(1.21 - 1.60)	(1.09 - 1.45)	(1.20 - 1.38)	(1.23 - 1.44)	(1.38 - 1.83)

95% CI in parentheses. All relative risks statistically significantly different from 1 at the 5% level.

Sensitivity Analyses

Robustness to Model Specifications

We explore the robustness of our results to model specifications by running logistic regressions with different independent variables. Our results that HIV is associated with higher levels of chronic disease onset is not sensitive to these variations.

Table S7. Logistic Regression Outcomes. 2-Year Odds ratios of Hypertension. HIV variable is defined by Cohort 1, ever diagnosed with HIV

Model	1	2	3
HIV/AIDS status at enrollment	1.132***	1.133***	1.139***
	(1.066 - 1.202)	(1.067 - 1.203)	(1.073 - 1.208)
Age at enrollment	1.031***	1.030***	1.027***
	(1.031 - 1.031)	(1.030 - 1.030)	(1.027 - 1.028)
Male	1.212***	1.194***	1.211***
	(1.205 - 1.220)	(1.187 - 1.202)	(1.204 - 1.219)
Black	1.131***	1.131***	1.155***
	(1.118 - 1.144)	(1.118 - 1.144)	(1.142 - 1.168)
Annual household income < 50K	1.155***	1.152***	1.205***
	(1.146 - 1.163)	(1.144 - 1.160)	(1.197 - 1.213)
Diabetes status at enrollment	0.951***		1.027***
	(0.942 - 0.961)		(1.017 - 1.036)
Stroke status at enrollment	1.005		1.001
	(0.968 - 1.044)		(0.964 - 1.039)
Cancer status at enrollment	0.871***		0.865***
	(0.857 - 0.885)		(0.851 - 0.879)
Lung disease status at enrollment	0.972***		1.046***
	(0.960 - 0.984)		(1.033 - 1.059)
Cardiovascular disease status at enrollment	0.821***		0.861***
	(0.812 - 0.830)		(0.852 - 0.870)
Cog. impairment and dementia status at enrollment	1.154***		1.148***
	(1.138 - 1.170)		(1.132 - 1.164)
Obesity status at enrollment	1.695***	1.668***	
	(1.682 - 1.709)	(1.655 - 1.681)	
Alcohol-related disease status at enrollment	1.605***	1.597***	
	(1.550 - 1.662)	(1.542 - 1.654)	
Substance abuse status at enrollment	1.267***	1.266***	
	(1.100 - 1.458)	(1.100 - 1.457)	
Smoking status at enrollment	1.443***	1.426***	
	(1.429 - 1.456)	(1.413 - 1.439)	
Observations	3,381,256	3,381,256	3,381,256

Robust standard errors in parentheses *** p<0.01, ** p<0.05, * p<0.1

Table S8. Logistic Regression Outcomes. 2-Year Odds ratios of Cognitive Impairment/Dementia. HIV variable is defined by Cohort 1, ever diagnosed with HIV.

Model	1	2	3
HIV/AIDS status at enrollment	1.662***	1.676***	1.721***
	(1.483 - 1.862)	(1.496 - 1.878)	(1.537 - 1.928)
Age at enrollment	1.095***	1.096***	1.088***
	(1.094 - 1.096)	(1.095 - 1.096)	(1.087 - 1.088)
Male	0.909***	0.938***	0.926***
	(0.898 - 0.920)	(0.927 - 0.949)	(0.915 - 0.938)
Black	0.956***	0.937***	0.974***
	(0.937 - 0.974)	(0.919 - 0.955)	(0.955 - 0.993)
Annual household income < 50K	1.218***	1.225***	1.271***
	(1.203 - 1.233)	(1.210 - 1.240)	(1.255 - 1.286)
Diabetes status at enrollment	1.094***		1.138***
	(1.079 - 1.109)		(1.122 - 1.153)
Hypertension status at enrollment	0.810***		0.831***
	(0.799 - 0.821)		(0.821 - 0.842)
Stroke status at enrollment	1.408***		1.413***
	(1.361 - 1.455)		(1.367 - 1.461)
Cancer status at enrollment	0.915***		0.911***
	(0.895 - 0.936)		(0.891 - 0.932)
Lung disease status at enrollment	1.007		1.112***
	(0.990 - 1.025)		(1.093 - 1.132)
Cardiovascular disease status at enrollment	1.248***		1.297***
	(1.231 - 1.266)		(1.279 - 1.315)
Obesity status at enrollment	1.424***	1.435***	
	(1.404 - 1.445)	(1.415 - 1.455)	
Alcohol-related disease status at enrollment	2.904***	2.935***	
	(2.770 - 3.045)	(2.800 - 3.077)	
Substance abuse status at enrollment	2.217***	2.240***	
	(1.817 - 2.706)	(1.836 - 2.734)	
Smoking status at enrollment	1.719***	1.743***	
	(1.691 - 1.748)	(1.715 - 1.772)	
Observations	5,907,482	5,907,482	5,907,482

Robust standard errors in parentheses. *** p<0.01, ** p<0.05, * p<0.1

Table S9. Logistic Regression Outcomes. 2-Year Odds ratios of Stroke. HIV variable is defined by Cohort 1, ever diagnosed with HIV

Model	1	2	3
HIV/AIDS status at enrollment	1.280***	1.457***	1.314***
	(1.163 - 1.408)	(1.326 - 1.602)	(1.195 - 1.446)
Age at enrollment	1.046***	1.070***	1.040***
	(1.046 - 1.047)	(1.069 - 1.070)	(1.039 - 1.040)
Male	1.039***	1.087***	1.058***
	(1.029 - 1.050)	(1.076 - 1.098)	(1.047 - 1.069)
Black	1.073***	1.103***	1.093***
	(1.055 - 1.090)	(1.085 - 1.120)	(1.075 - 1.110)
Annual household income < 50K	1.183***	1.238***	1.237***
	(1.171 - 1.196)	(1.224 - 1.251)	(1.224 - 1.251)
Diabetes status at enrollment	1.215***		1.252***
	(1.201 - 1.229)		(1.238 - 1.266)
Hypertension status at enrollment	0.995		1.021***
	(0.983 - 1.006)		(1.009 - 1.033)
Cancer status at enrollment	0.926***		0.923***
	(0.908 - 0.944)		(0.905 - 0.942)
Lung disease status at enrollment	1.014*		1.124***
	(1.000 - 1.030)		(1.108 - 1.140)
Cardiovascular disease status at enrollment	1.567***		1.629***
	(1.548 - 1.586)		(1.610 - 1.649)
Cog. impairment and dementia status at enrollment	3.511***		3.526***
	(3.464 - 3.558)		(3.479 - 3.574)
Obesity status at enrollment	1.332***	1.440***	
	(1.315 - 1.348)	(1.423 - 1.458)	
Alcohol-related disease status at enrollment	1.583***	1.873***	
	(1.511 - 1.659)	(1.789 - 1.960)	
Substance abuse status at enrollment	1.572***	2.014***	
	(1.334 - 1.852)	(1.715 - 2.365)	
Smoking status at enrollment	1.766***	1.882***	
	(1.742 - 1.791)	(1.857 - 1.907)	
Observations	6,215,432	6,215,432	6,215,432

Robust standard errors in parentheses *** p<0.01, ** p<0.05, * p<0.1

Table S10. Logistic Regression Outcomes. 2-Year Odds ratios of Cancer. HIV variable is defined by Cohort 1, ever diagnosed with HIV

Model	1	2	3
HIV/AIDS status at enrollment	1.442***	1.441***	1.470***
	(1.314 - 1.582)	(1.313 - 1.580)	(1.340 - 1.613)
Age at enrollment	1.048***	1.045***	1.042***
	(1.047 - 1.048)	(1.044 - 1.045)	(1.041 - 1.042)
Male	1.236***	1.226***	1.256***
	(1.223 - 1.250)	(1.213 - 1.239)	(1.242 - 1.269)
Black	1.042***	1.005	1.061***
	(1.024 - 1.060)	(0.987 - 1.022)	(1.043 - 1.080)
Annual household income < 50K	1.056***	1.050***	1.109***
	(1.044 - 1.068)	(1.038 - 1.062)	(1.096 - 1.121)
Diabetes status at enrollment	0.994		1.027***
	(0.981 - 1.007)		(1.014 - 1.040)
Hypertension status at enrollment	0.788***		0.809***
	(0.779 - 0.798)		(0.800 - 0.819)
Stroke status at enrollment	0.853***		0.855***
	(0.824 - 0.884)		(0.825 - 0.886)
Lung disease status at enrollment	1.121***		1.244***
	(1.104 - 1.139)		(1.225 - 1.264)
Cardiovascular disease status at enrollment	0.985**		1.025***
	(0.972 - 0.998)		(1.012 - 1.039)
Cog. impairment and dementia status at enrollment	1.074***		1.072***
	(1.054 - 1.094)		(1.052 - 1.092)
Obesity status at enrollment	1.346***	1.292***	
	(1.328 - 1.363)	(1.275 - 1.308)	
Alcohol-related disease status at enrollment	1.321***	1.315***	
	(1.252 - 1.394)	(1.246 - 1.387)	
Substance abuse status at enrollment	0.725**	0.716**	
	(0.562 - 0.936)	(0.554 - 0.923)	
Smoking status at enrollment	1.779***	1.787***	
	(1.754 - 1.805)	(1.762 - 1.812)	
Observations	5,981,854	5,981,854	5,981,854

Robust standard errors in parentheses *** p<0.01, ** p<0.05, * p<0.1

Table S11. Logistic Regression Outcomes. 2-Year Odds ratios of Lung Disease. HIV variable is defined by Cohort 1, ever diagnosed with HIV

Model	1	2	3
HIV/AIDS status at enrollment	1.269***	1.292***	1.315***
	(1.193 - 1.350)	(1.215 - 1.374)	(1.237 - 1.397)
Age at enrollment	1.021***	1.024***	1.012***
	(1.020 - 1.021)	(1.024 - 1.025)	(1.011 - 1.012)
Male	0.906***	0.926***	0.936***
	(0.900 - 0.912)	(0.919 - 0.932)	(0.929 - 0.942)
Black	0.873***	0.863***	0.905***
	(0.863 - 0.883)	(0.854 - 0.873)	(0.895 - 0.915)
Annual household income < 50K	1.214***	1.221***	1.322***
	(1.205 - 1.222)	(1.212 - 1.230)	(1.313 - 1.332)
Diabetes status at enrollment	1.045***		1.100***
	(1.036 - 1.053)		(1.091 - 1.109)
Hypertension status at enrollment	0.880***		0.922***
	(0.874 - 0.887)		(0.915 - 0.929)
Stroke status at enrollment	0.929***		0.935***
	(0.908 - 0.950)		(0.915 - 0.957)
Cancer status at enrollment	1.010		1.001
	(0.996 - 1.024)		(0.987 - 1.015)
Cardiovascular disease	1.247***		1.339***
	(1.236 - 1.257)		(1.328 - 1.351)
Cog. impairment and dementia status at enrollment	1.325***		1.326***
	(1.309 - 1.341)		(1.311 - 1.342)
Obesity status at enrollment	1.560***	1.566***	
	(1.547 - 1.572)	(1.554 - 1.578)	
Alcohol-related disease status at enrollment	1.701***	1.746***	
	(1.648 - 1.756)	(1.692 - 1.803)	
Substance abuse status at enrollment	0.951	0.982	
	(0.828 - 1.092)	(0.855 - 1.127)	
Smoking status at enrollment	2.644***	2.677***	
	(2.621 - 2.666)	(2.655 - 2.700)	
Observations	5,658,282	5,658,282	5,658,282

Robust standard errors in parentheses *** p<0.01, ** p<0.05, * p<0.1

Table S12. Logistic Regression Outcomes. 2-Year Odds ratios of Cardiovascular Disease. HIV variable is defined by Cohort 1, ever diagnosed with HIV

Model	1	2	3
HIV/AIDS status at enrollment	1.300***	1.322***	1.325***
	(1.203 - 1.404)	(1.225 - 1.428)	(1.228 - 1.430)
Age at enrollment	1.056***	1.059***	1.047***
	(1.056 - 1.057)	(1.058 - 1.059)	(1.047 - 1.048)
Male	1.323***	1.324***	1.334***
	(1.312 - 1.334)	(1.313 - 1.335)	(1.323 - 1.345)
Black	0.964***	0.985**	0.988*
	(0.951 - 0.978)	(0.972 - 0.999)	(0.975 - 1.002)
Annual household income < 50K	1.176***	1.197***	1.258***
	(1.166 - 1.187)	(1.187 - 1.208)	(1.247 - 1.269)
Diabetes status at enrollment	1.221***		1.327***
	(1.209 - 1.233)		(1.314 - 1.340)
Hypertension status at enrollment	1.012**		1.077***
	(1.003 - 1.021)		(1.068 - 1.087)
Stroke status at enrollment	1.282***		1.290***
	(1.242 - 1.322)		(1.250 - 1.330)
Cancer status at enrollment	0.913***		0.909***
	(0.898 - 0.930)		(0.893 - 0.925)
Lung disease status at enrollment	1.174***		1.354***
	(1.159 - 1.190)		(1.337 - 1.371)
Cog. impairment and dementia status at enrollment	1.138***		1.136***
	(1.120 - 1.156)		(1.118 - 1.154)
Obesity status at enrollment	1.975***	2.058***	
	(1.957 - 1.994)	(2.039 - 2.077)	
Alcohol-related disease status at enrollment	2.046***	2.100***	
	(1.971 - 2.125)	(2.023 - 2.180)	
Substance abuse status at enrollment	1.209**	1.253***	
	(1.028 - 1.421)	(1.066 - 1.473)	
Smoking status at enrollment	2.023***	2.063***	
	(2.000 - 2.045)	(2.040 - 2.085)	
Observations	5,027,839	5,027,839	5,027,839

Robust standard errors in parentheses. *** p<0.01, ** p<0.05, * p<0.1

Table S13. Logistic Regression Outcomes. 2-Year Odds ratios of Diabetes. HIV variable is defined by Cohort 1, ever diagnosed with HIV

Model	1	2	3
HIV/AIDS status at enrollment	1.303***	1.313***	1.280***
	(1.194 - 1.422)	(1.203 - 1.433)	(1.174 - 1.397)
Age at enrollment	1.017***	1.021***	1.009***
	(1.016 - 1.018)	(1.020 - 1.021)	(1.008 - 1.009)
Male	1.202***	1.226***	1.181***
	(1.190 - 1.214)	(1.214 - 1.238)	(1.169 - 1.192)
Black	1.283***	1.300***	1.329***
	(1.263 - 1.303)	(1.280 - 1.320)	(1.309 - 1.350)
Annual household income < 50K	1.225***	1.236***	1.287***
	(1.212 - 1.239)	(1.223 - 1.250)	(1.273 - 1.301)
Hypertension status at enrollment	1.125***		1.244***
	(1.112 - 1.137)		(1.231 - 1.258)
Stroke status at enrollment	0.969*		0.949***
	(0.936 - 1.003)		(0.916 - 0.982)
Cancer status at enrollment	0.858***		0.852***
	(0.840 - 0.877)		(0.833 - 0.870)
Lung disease status at enrollment	1.024***		1.117***
	(1.008 - 1.040)		(1.100 - 1.135)
Cardiovascular disease status at enrollment	1.224***		1.305***
	(1.208 - 1.240)		(1.288 - 1.322)
Cog. impairment and dementia status at enrollment	1.000		0.977**
	(0.980 - 1.020)		(0.958 - 0.997)
Obesity status at enrollment	2.504***	2.584***	
	(2.477 - 2.531)	(2.557 - 2.612)	
Alcohol-related disease status at enrollment	1.826***	1.861***	
	(1.744 - 1.912)	(1.777 - 1.948)	
Substance abuse status at enrollment	0.981	1.014	
	(0.804 - 1.195)	(0.832 - 1.235)	
Smoking status at enrollment	1.387***	1.422***	
	(1.368 - 1.406)	(1.403 - 1.441)	
Observations	4,829,266	4,829,266	4,829,266

Robust standard errors in parentheses *** p<0.01, ** p<0.05, * p<0.1

Additional Sensitivity Analyses of Cohort 3 Outcomes

Year of HIV diagnosis
The regression analysis was repeated for individuals in Cohort 3 diagnosed with HIV/AIDS before and after 2011, as ART has changed dramatically since 2007 and there may be temporal effects. We find that the results are similar to those in the main manuscript.

Table S14. HIV+ population used: Individuals in Cohort 3 (diagnosed with HIV after two years from enrollment) diagnosed with HIV/AIDS before 2011

	Diabetes	Hypertension	Stroke	Cancer	Lung Diseases	Cardiovascular Diseases	Dementia
HIV/AIDS status at enrollment	3.64	2.75	2.82	2.40	2.76	3.86	3.17
	(3.06 - 4.34)	(2.30 - 3.29)	(2.31 - 3.44)	(1.97 - 2.93)	(2.38 - 3.20)	(3.25 - 4.58)	(2.49 - 4.05)
Age at enrollment	1.02	1.05	1.05	1.05	1.03	1.07	1.11
	(1.02 - 1.02)	(1.05 - 1.05)	(1.05 - 1.05)	(1.05 - 1.05)	(1.03 - 1.03)	(1.07 - 1.07)	(1.11 - 1.11)
Male	1.23	1.21	0.99	1.17	0.85	1.38	0.89
	(1.22 - 1.24)	(1.20 - 1.21)	(0.98 - 0.99)	(1.17 - 1.18)	(0.84 - 0.85)	(1.37 - 1.38)	(0.88 - 0.89)
Black	1.29	1.40	1.11	1.07	0.86	0.95	0.99**
	(1.28 - 1.30)	(1.39 - 1.42)	(1.09 - 1.12)	(1.06 - 1.08)	(0.86 - 0.87)	(0.95 - 0.96)	(0.98 - 1.00)
Annual household income <50,000	1.22	1.22	1.13	0.99	1.21	1.14	1.15
	(1.21 - 1.23)	(1.21 - 1.22)	(1.12 - 1.13)	(0.98 - 0.99)	(1.20 - 1.21)	(1.14 - 1.15)	(1.14 - 1.16)
Diabetes status at enrollment		2.73	1.22	*1.00*	1.07	1.22	1.06
		(2.71 - 2.75)	(1.21 - 1.23)	(0.99 - 1.00)	(1.06 - 1.07)	(1.22 - 1.23)	(1.05 - 1.07)
Hypertension status at enrollment	1.03		0.91	0.72	0.80	0.93	0.74
	(1.02 - 1.03)		(0.90 - 0.92)	(0.71 - 0.72)	(0.80 - 0.81)	(0.92 - 0.93)	(0.74 - 0.75)
Stroke status at enrollment	0.91	0.67		0.80	0.86	1.23	1.38
	(0.89 - 0.93)	(0.66 - 0.69)		(0.78 - 0.82)	(0.85 - 0.87)	(1.21 - 1.26)	(1.35 - 1.41)
Cancer status at enrollment	0.82	0.69	0.89		*1.01*	0.88	0.88
	(0.81 - 0.83)	(0.69 - 0.70)	(0.88 - 0.90)		(1.00 - 1.02)	(0.87 - 0.89)	(0.86 - 0.89)
Lung diseases at enrollment	0.96	0.75	0.94	1.04		1.13	0.94

	(0.95 - 0.97)	(0.74 - 0.76)	(0.94 - 0.95)	(1.03 - 1.05)		(1.12 - 1.14)	(0.93 - 0.95)
Cardiovascular diseases status at enrollment	1.19	2.64	1.97	1.10	1.55		1.18
	(1.18 - 1.20)	(2.62 - 2.66)	(1.95 - 1.98)	(1.09 - 1.10)	(1.54 - 1.55)		(1.17 - 1.19)
Dementia status at enrollment	1.06	1.26	3.01	0.95	1.23	1.45	
	(1.05 - 1.07)	(1.24 - 1.27)	(2.99 - 3.04)	(0.94 - 0.96)	(1.22 - 1.24)	(1.43 - 1.46)	
Obesity at enrollment	3.01	3.05	1.52	1.49	1.86	2.43	1.62
	(2.99 - 3.03)	(3.03 - 3.07)	(1.51 - 1.53)	(1.48 - 1.50)	(1.85 - 1.87)	(2.41 - 2.44)	(1.61 - 1.64)
Alcohol-related diseases at enrollment	1.95	2.52	1.76	1.36	1.90	2.37	3.14
	(1.89 - 2.01)	(2.44 - 2.61)	(1.71 - 1.82)	(1.32 - 1.41)	(1.86 - 1.94)	(2.31 - 2.44)	(3.04 - 3.24)
Substance Abuse at enrollment	1.00	1.54	1.76	0.84	*1.05*	1.29	2.59
	(0.88 - 1.14)	(1.36 - 1.74)	(1.58 - 1.96)	(0.73 - 0.97)	(0.96 - 1.15)	(1.15 - 1.44)	(2.28 - 2.95)
Smoking status at enrollment	1.46	1.81	1.93	1.90	3.44	2.31	1.85
	(1.45 - 1.47)	(1.80 - 1.82)	(1.91 - 1.95)	(1.89 - 1.92)	(3.42 - 3.46)	(2.30 - 2.33)	(1.84 - 1.87)
Constant	0.01	0.02	0.00	0.00	0.03	0.00	0.00
	(0.01 - 0.01)	(0.02 - 0.02)	(0.00 - 0.00)	(0.00 - 0.00)	(0.03 - 0.03)	(0.00 - 0.00)	(0.00 - 0.00)
Observations	5,724,270	4,037,359	7,358,402	7,088,435	6,697,859	5,975,517	7,008,298

Robust 95% CI in parentheses. All OD were statistically significantly different from 1 at the 5% level except those in italics.

Table S15. HIV+ Population Used: Individuals in Cohort 3 (diagnosed with HIV after two years from enrollment) diagnosed with HIV/AIDS in/after 2011

	Diabetes	Hypertension	Stroke	Cancer	Lung Diseases	Cardiovascular Diseases	Dementia
HIV/AIDS status at enrollment	4.25	3.02	2.68	2.16	2.64	4.00	3.90
	(3.81 - 4.73)	(2.66 - 3.42)	(2.39 - 3.01)	(1.92 - 2.44)	(2.40 - 2.90)	(3.61 - 4.42)	(3.44 - 4.43)
Age at enrollment	1.02	1.05	1.05	1.05	1.03	1.07	1.11
	(1.02 - 1.02)	(1.05 - 1.05)	(1.05 - 1.05)	(1.05 - 1.05)	(1.03 - 1.03)	(1.07 - 1.07)	(1.11 - 1.11)
Male	1.23	1.21	0.99	1.17	0.85	1.38	0.89
	(1.22 - 1.24)	(1.20 - 1.21)	(0.98 - 0.99)	(1.17 - 1.18)	(0.84 - 0.85)	(1.37 - 1.38)	(0.88 - 0.89)
Black	1.29	1.40	1.11	1.07	0.86	0.95	0.99
	(1.28 - 1.30)	(1.39 - 1.42)	(1.09 - 1.12)	(1.06 - 1.08)	(0.86 - 0.87)	(0.95 - 0.96)	(0.98 - 1.00)
Annual household income <50,000	1.22	1.22	1.13	0.99	1.21	1.14	1.15
	(1.21 - 1.23)	(1.21 - 1.22)	(1.12 - 1.13)	(0.98 - 0.99)	(1.20 - 1.21)	(1.14 - 1.15)	(1.14 - 1.16)
Diabetes status at enrollment		2.73	1.22	*1.00*	1.07	1.22	1.06
		(2.71 - 2.75)	(1.21 - 1.23)	(0.99 - 1.00)	(1.06 - 1.07)	(1.22 - 1.23)	(1.05 - 1.07)
Hypertension status at enrollment	1.03		0.91	0.72	0.80	0.93	0.74
	(1.02 - 1.03)		(0.90 - 0.92)	(0.71 - 0.72)	(0.80 - 0.81)	(0.92 - 0.93)	(0.74 - 0.75)
Stroke status at enrollment	0.91	0.67		0.80	0.86	1.23	1.38
	(0.89 - 0.93)	(0.66 - 0.69)		(0.78 - 0.82)	(0.85 - 0.87)	(1.21 - 1.26)	(1.35 - 1.41)
Cancer status at enrollment	0.82	0.69	0.89		*1.01*	0.88	0.88
	(0.81 - 0.83)	(0.69 - 0.70)	(0.88 - 0.91)		(1.00 - 1.02)	(0.87 - 0.89)	(0.86 - 0.89)
Lung diseases at enrollment	0.96	0.75	0.94	1.04		1.13	0.94
	(0.95 - 0.97)	(0.74 - 0.76)	(0.94 - 0.95)	(1.03 - 1.05)		(1.12 - 1.14)	(0.93 - 0.95)
Cardiovascular diseases status at enrollment	1.19	2.64	1.97	1.10	1.55		1.18
	(1.18 - 1.20)	(2.62 - 2.66)	(1.95 - 1.98)	(1.09 - 1.10)	(1.54 - 1.55)		(1.17 - 1.19)
Dementia status at enrollment	1.06	1.26	3.01	0.95	1.23	1.45	
	(1.05 - 1.07)	(1.24 - 1.27)	(2.99 - 3.04)	(0.94 - 0.96)	(1.22 - 1.24)	(1.43 - 1.46)	

Obesity at enrollment	3.01	3.05	1.52	1.49	1.86	2.43	1.62
	(2.98 - 3.03)	(3.03 - 3.07)	(1.50 - 1.53)	(1.48 - 1.50)	(1.85 - 1.87)	(2.41 - 2.44)	(1.61 - 1.64)
Alcohol-related diseases at enrollment	1.95	2.52	1.76	1.36	1.90	2.38	3.14
	(1.89 - 2.01)	(2.44 - 2.61)	(1.71 - 1.82)	(1.32 - 1.41)	(1.86 - 1.94)	(2.31 - 2.44)	(3.05 - 3.24)
Substance Abuse at enrollment	1.01	1.55	1.76	0.84	1.05	1.29	2.60
	(0.89 - 1.15)	(1.37 - 1.75)	(1.59 - 1.96)	(0.73 - 0.97)	(0.96 - 1.16)	(1.16 - 1.44)	(2.29 - 2.95)
Smoking status at enrollment	1.46	1.81	1.93	1.90	3.44	2.31	1.85
	(1.45 - 1.47)	(1.80 - 1.82)	(1.91 - 1.95)	(1.89 - 1.92)	(3.42 - 3.46)	(2.30 - 2.33)	(1.84 - 1.87)
Constant	0.01	0.02	0.00	0.00	0.03	0.00	0.00
	(0.01 - 0.01)	(0.02 - 0.02)	(0.00 - 0.00)	(0.00 - 0.00)	(0.03 - 0.03)	(0.00 - 0.00)	(0.00 - 0.00)
Observations	5,725,291	4,038,085	7,360,053	7,090,035	6,699,249	5,976,788	7,009,824

Robust 95% CI in parentheses. All OD were statistically significantly different from 1 at the 5% level except those in italics.

Cohort 3 Analysis: Age of HIV diagnosis

Cohort 3 HIV+ individuals may be of particular interest, as their dates of HIV diagnosis are observable. Summary statistics for this cohort by age are provided in the table below.

Table S16. Cohort 3 (diagnosed with HIV after two years from enrollment) summary statistics by age group (%)

Age	50-59		60-69		70-79		>=80	
HIV status	HIV -	HIV +	HIV -	HIV +	HIV -	HIV +	HIV -	HIV +
	%	%	%	%	%	%	%	%
Male	48	64.9	46.3	59.9	43.5	49.6	38.6	40.9
White	77.1	56.3	76.2	55.4	75.3	56.7	78.3	58.6
Black	10.3	25.9	10.5	27.4	10.6	23.6	10.1	15.7
Hispanic	9.3	15	9.7	14.9	10.4	16.5	8.6	22.7
Education level < high school	0.5	1.1	0.7	1.2	0.9	1.7	0.7	0
Annual household income < 50000	22.2	35.3	32.8	44.9	46.5	52.8	54.3	59.7
Diabetes status at enrollment	16.2	25.1	26.9	38.5	31.5	40.9	32.7	47.5
Hypertension status at enrollment	31.5	38.3	49.1	55.7	60.3	61	71.1	74.7
Stroke status at enrollment	0.9	2.4	2	3.2	4.1	5.8	6.9	4.5
Cancer status at enrollment	2.7	2.5	5.7	5.5	9.3	9.5	11.1	12.1
Lung diseases status at enrollment	7.9	12.2	11.5	16.1	14.8	17.6	17.4	20.2
Cardiovascular diseases status at enrollment	9.9	15.9	20.1	26.9	33.5	41	48.7	51.5
Dementia status at enrollment	1.9	4.2	4.5	10.6	14.7	24.5	35.1	48.5
Obesity status at enrollment	18.6	27.6	20.1	32.1	15.6	23.5	7.9	13.1
Alcohol-related status at enrollment	0.7	3.8	0.7	3.2	0.6	2.1	0.3	1
Substance abuse status at enrollment	0.1	0.5	0.1	0.4	0	0.1	0	0
Smoking status at enrollment	13.6	26.2	12.6	22.3	9.3	15.6	4.7	8.6

We include the results of a sensitivity analysis of Cohort 3 that includes age of diagnosis in addition to the other covariates below. These results suggest that HIV patients have higher odds ratios of acquiring chronic illness at every age. The table reports the odds ratios associated with the HIV variable.

Independent variables used include demographic variables, comorbidities, and behavioral factors. The demographic variables are: HIV status, male, black, and annual household income < 50K. The comorbidity independent variables are: diabetes status at enrollment, stroke status at enrollment, cancer status at enrollment, lung disease status at enrollment, cardiovascular disease status at enrollment, cog. impairment and dementia status at enrollment. The behavioral independent variables are: obesity status at enrollment, alcohol-related disease status at enrollment, substance abuse status at enrollment, and smoking status at enrollment. Model 1 uses all independent variables, model 2 excludes variables on comorbidities, and model 3 excludes behavioral variables.

Table S17. HIV odds ratios by age group for Cohort 3 (diagnosed with HIV after two years from enrollment) compared with HIV- enrollees

Age group	Diabetes	Hypertension	Stroke	Cancer	Lung diseases	Cardiovascular diseases	Dementia
Model Specification 1							
50-59	3.87<br>(3.31 - 4.53)	2.39<br>(2.06 - 2.76)	3.59<br>(2.95 - 4.37)	2.42<br>(1.98 - 2.94)	2.78<br>(2.42 - 3.20)	4.08<br>(3.50 - 4.75)	4.95<br>(3.90 - 6.29)
60-69	4.39<br>(3.76 - 5.13)	3.37<br>(2.81 - 4.03)	2.75<br>(2.31 - 3.27)	2.51<br>(2.12 - 2.98)	2.67<br>(2.32 - 3.06)	3.73<br>(3.22 - 4.32)	3.94<br>(3.27 - 4.76)
70-79	4.24<br>(3.46 - 5.20)	3.22<br>(2.35 - 4.42)	2.59<br>(2.16 - 3.12)	1.92<br>(1.56 - 2.36)	2.62<br>(2.22 - 3.09)	4.35<br>(3.60 - 5.26)	3.14<br>(2.57 - 3.84)
>=80	3.51<br>(2.64 - 4.67)	8.57<br>(4.31 - 17.02)	2.92<br>(2.33 - 3.65)	1.84<br>(1.40 - 2.41)	2.76<br>(2.20 - 3.47)	4.39<br>(3.32 - 5.80)	3.41<br>(2.61 - 4.46)
Model Specification 2							
50-59	3.94<br>(3.39 - 4.58)	2.49<br>(2.16 - 2.88)	3.38<br>(2.79 - 4.11)	2.58<br>(2.13 - 3.14)	2.90<br>(2.55 - 3.31)	4.31<br>(3.73 - 4.98)	5.32<br>(4.21 - 6.72)
60-69	4.87<br>(4.20 - 5.65)	3.61<br>(3.02 - 4.31)	2.95<br>(2.49 - 3.50)	2.78<br>(2.35 - 3.29)	3.00<br>(2.64 - 3.41)	4.20<br>(3.66 - 4.83)	4.50<br>(3.73 - 5.43)
70-79	4.63<br>(3.81 - 5.63)	3.58<br>(2.62 - 4.89)	2.79<br>(2.31 - 3.37)	2.14<br>(1.74 - 2.63)	2.96<br>(2.52 - 3.47)	4.77<br>(3.99 - 5.70)	3.57<br>(2.93 - 4.34)
>=80	3.53<br>(2.68 - 4.66)	8.50<br>(4.32 - 16.70)	2.71<br>(2.12 - 3.45)	1.86<br>(1.42 - 2.43)	2.71<br>(2.16 - 3.39)	4.41<br>(3.34 - 5.80)	3.51<br>(2.71 - 4.55)
Model Specification 3							

50-59	3.84 (3.28 - 4.49)	2.40 (2.07 - 2.79)	3.17 (2.60 - 3.87)	2.42 (1.99 - 2.95)	2.72 (2.37 - 3.13)	3.99 (3.42 - 4.64)	4.92 (3.88 - 6.24)
60-69	4.40 (3.77 - 5.15)	3.37 (2.82 - 4.03)	2.63 (2.20 - 3.14)	2.52 (2.12 - 2.99)	2.66 (2.32 - 3.05)	3.68 (3.18 - 4.27)	3.95 (3.27 - 4.77)
70-79	4.23 (3.45 - 5.19)	3.21 (2.32 - 4.43)	2.51 (2.07 - 3.05)	1.93 (1.57 - 2.37)	2.61 (2.21 - 3.08)	4.28 (3.54 - 5.18)	3.15 (2.58 - 3.85)
>=80	3.51 (2.64 - 4.67)	8.43 (4.24 - 16.74)	2.64 (2.07 - 3.36)	1.80 (1.37 - 2.37)	2.64 (2.09 - 3.32)	4.24 (3.19 - 5.64)	N/A^{++}

Robust 95% CI in parentheses. All odds ratios statistically significantly different from 1 at the 5% level.

N/A^{++}: There is no substance abuse data among PLWHA without dementia aged >= 80.
